# An international survey of the relatives and friends of electroconvulsive therapy recipients

**DOI:** 10.1111/papt.70062

**Published:** 2026-04-22

**Authors:** Christopher Harrop, Sue Cunliffe, Sarah Price Hancock, Lucy Johnstone, Lisa Morrison, John Read

**Affiliations:** ^1^ Independent Scholar Woking UK; ^2^ Independent Scholar Worcester UK; ^3^ Independent Scholar San Diego USA; ^4^ Independent Scholar Bristol UK; ^5^ Independent Scholar Belfast UK; ^6^ Department of Psychology and Human Development University of East London London UK

**Keywords:** adverse effects, electroconvulsive therapy; relatives, family, friends, informed consent

## Abstract

**Objectives:**

This study aimed to address the paucity of studies of the relatives and friends of electroconvulsive therapy (ECT) patients.

**Methods:**

A total of 1144 people responded to an online survey.

**Results:**

The respondents included 286 relatives and friends of ECT recipients, from 22 countries. 45% reported improvement in the problem for which ECT was prescribed, and 42% said it made the problem worse. 61% thought ECT had made ‘overall quality of life’ worse and 32% reported improvement. On three measures of memory, between 51% and 73% reported memory loss in their relative or friend (mostly lasting at least 3 years), and 8%–11% reported memory improvement. Twenty one of 25 other adverse effects from ECT were reported by 50% or more of the relatives/friends, including: Difficulty concentrating (79%), Fatigue (73%), Emotional blunting (73%), Loss of independence (72%) and Relationship problems (70%). 34% believed ECT had caused brain damage. When asked, ‘Would you want to have ECT yourself, if a psychiatrist thought you needed it?’ 72% said no. Two open questions, about impact on the patient and on the relative/friend, both elicited about three times as many negative impacts as positive ones.

**Conclusions:**

The responses of the relatives/friends are broadly similar to ECT recipients' responses to the same survey, reported elsewhere, which tends to support the accuracy of the ECT recipients' responses. ECT recipients and their families need a more detailed and accurate picture of possible benefits and harms if they are to give fully informed consent.

## INTRODUCTION

Electroconvulsive therapy (ECT) involves passing electricity through the brain, under general anesthesia, to cause a generalized, tonic–clonic seizure, typically between six and 12 times, over several weeks. Despite the decline in usage over several decades, about one million people worldwide still receive ECT annually (Leiknes et al., 2012), predominantly women and older people, including about 2500 in England (Read et al., 2018, 2021).

An international review found large variation between continents, countries and regions in utilization rates and clinical practice (Leiknes et al., 2012). A subsequent review (Wingralek et al., 2022) also found huge variation in ECT patients per 100,000 of the population between the highest using countries (United States —51, Belgium—47, Sweden—41 and Australia—38) and the lowest (Germany—2.5, Poland—1.3, Latvia—0.5). Two audits in England found 12‐fold (Read et al., 2018) and 47‐fold (Read et al., 2021) differences in rates of usage per population between the lowest and highest using areas. There is also significant regional variation within the United States (Peltzman et al., 2020).

Although initially introduced in 1938 to treat people diagnosed with ‘schizophrenia’, ECT soon became predominantly used for depression, although it is still also used, in smaller numbers, on people diagnosed with ‘schizophrenia’, ‘bipolar disorder’ and ‘catatonia’ (Leiknes et al., 2012). ECT is often considered a gold standard of care for people who have not responded to other treatments (Goldberg, 2022). ECT, nevertheless, remains controversial (Funk et al., 2025; Read, Cunliffe, et al., 2019, 2026b). One meta‐analysis of ECT research found views varying from ‘probably ineffective but certainly causes brain damage… through to those who think it is the most effective treatment in psychiatry and completely safe’ (UK ECT Review Group, 2003, p. 799).

### Efficacy

There have only ever been 11 placebo‐controlled studies of ECT for depression, and those studies were all small (between 8 and 77 participants), flawed and are all now at least 40 years old (Read & Bentall, 2010; Read, Kirsch, & McGrath, 2019). (Placebo ECT, or sham ECT [SECT], involves administering the general anaesthetic but not the electric shock). Furthermore, all 11 studies involved sine machines, which are no longer in use. A 2019 review (co‐authored by Dr. Irving Kirsch, Associate Director of Harvard Medical School's Programme in Placebo Studies) found that none of the 11 studies were genuinely double‐blind. Only four reported any patients' self‐ratings and none assessed Quality of Life. Four of the eleven found ECT superior to SECT at the end of treatment and five found no difference. The other two found that psychiatrists reported a difference, but patients did not. Neither of the two high‐quality studies that reported data at 1‐ or 6‐month post‐treatment produced a significant difference between ECT and SECT. When pooled, they produced a small effect size (0.017) in favour of SECT (Read, Kirsch, & McGrath, 2019).

In the absence of placebo‐controlled studies, researchers have turned to comparing ECT to other treatments for depression. A review of all such non‐placebo studies between 2009 and 2016 (Read & Arnold, 2017) found little evidence that ECT is effective for depression, primarily because most of the studies either maintained ECT participants on medication or produced no meaningful follow‐up data beyond the end of treatment, or both.

More recently, a randomized clinical trial for depression found response rates, at the end of treatment, of 55% for ketamine and 41% for ECT (Anand et al., 2023). A secondary analysis found that ketamine outperformed ECT for outpatients with ‘moderately severe or severe depression’ (Jha et al., 2024). For inpatients with ‘very severe depression’, ECT produced better results than ketamine early in the course of treatments but that difference disappeared by the end of the course. The study could not establish whether either treatment was better than placebo, because, like all ECT for depression studies since 1985, there was no placebo group. Also, like most ECT studies, there was no follow‐up beyond the last ECT.

There is also little evidence that ECT is effective for people diagnosed with ‘schizophrenia’. A 2019 analysis of Cochrane reviews concluded that ‘in spite of seven decades of clinical use of ECT for people with schizophrenia, there still is a lack of strong and adequate evidence regarding its effectiveness’ (Shokraneh et al., 2019). There have been two placebo‐controlled studies this century (Melzer‐Ribeiro et al., 2024; Ukpong et al., 2002). Neither found a difference between ECT and SECT, either at the end of treatment, or at follow‐up.

There is no consensus on how ECT works for those who do experience some short‐term benefit. Clearly, as we have noted above, placebo effects are at work (Melzer‐Ribeiro et al., 2024; Read & Bentall, 2010; Read, Kirsch, & McGrath, 2019). A review of over 100 human and animal brain studies led to the additional hypotheses that ECT affects the brain like severe stress or brain trauma, activating the HPA axis and the dopamine system and compromising frontotemporal functions. The temporary mild euphoria associated with brain trauma can be mistaken for lasting improvement in mood (Fosse & Read, 2013).

### ECT and relatives

This study reports the experiences of relatives and friends of ECT recipients. A recent systematic review (Boone et al., 2025) identified 29 studies of carers' attitudes, experiences or knowledge regarding ECT. About half (15) were in Asia, mostly India (9) and China (5), with two each from the United States and the United Kingdom. Most (17) had samples of 50 or fewer, and only two, both in China, had samples larger than 100 (Li et al., 2016; Zhang et al., 2018). (An Indian study (Grover et al., 2011) was accurately reported by the review as including 283 relatives overall, but only 77 were relatives of ECT recipients.) The review concluded that caregivers were generally satisfied with ECT but wanted more information on adverse effects. Memory disturbances were reported to be the most frequent and severe adverse effect. The review also noted the low methodological quality of the studies.

The only study (of 29) assessed as high quality by Boone et al. (2025) involved interviews with seven relatives in the United States (Smith et al., 2009), who reported mixed experiences. While some reported some positive outcomes, all described memory loss, which they felt negatively impacted quality of life. Some also reported being poorly informed of the risks of ECT. Some felt, nevertheless, that ECT had been the right decision for them. Others were angry at having agreed to ECT.

The only study conducted in the United Kingdom in the last 30 years (Guruvaiah et al., 2017) was rated as low quality by Boone et al. (2025). Psychiatrists had interviewed 27 carers and concluded that most felt that ECT was beneficial, but only half reported receiving any written information on ECT. Some said in the future they would only consider ECT as a last resort because of memory impairment.

The largest study to date (also rated as low quality by Boone et al., 2025) involved 210 relatives in Beijing (Li et al., 2016). It found, again, that relatives did not receive adequate information before ECT. Nevertheless, relatives had generally positive attitudes towards ECT.

Boone et al. (2025) did not include a mention of the issue by the Royal College of Psychiatrists' (2016) ECT Accreditation Service (ECTAS), perhaps because it was so brief. Every ECT clinic in the United Kingdom had been asked to distribute questionnaires to carers (Royal College of Psychiatrists, 2016). Only 36 carers returned the questionnaire, and the only report of the results was that carers were 'generally pleased' with the care provided by the clinic, with no mention of the positive or negative effects of the actual treatment. Subsequent ECTAS reports have not reported any data relating to relatives' perspectives.

Boone et al. (2025) recommended that future research should prioritize qualitative methods over quantitative ones, to gain a deeper understanding of caregivers' views. The current study addresses this call for qualitative methods, by including several open questions inviting written responses (see the Methods section). It is the largest study to date and the first international study. We are not aware of any studies reporting the views of the friends of ECT recipients.

The primary objective of this paper is to explore the perspectives of relatives and friends of ECT recipients on the positive and negative effects of ECT, including effects on their relationships with recipients and on themselves.

## METHODS

This project used the same methods as online surveys about other psychiatric treatments (Cartwright et al., 2016; Horowitz et al., 2025; Larsen‐Barr et al., 2018; Moncrieff et al., 2024; Read et al., 2014, 2017; Read, Lewis, et al., 2023; Read & Sacia, 2020; Read & Williams, 2019). It was approved by the Ethics and Integrity Sub‐Committee of the University of East London (ETH2324‐0053). Informed consent was given by ticking a box to indicate that the Participant Information Sheet had been read and agreed with before access to the survey questions.

### Instrument

A questionnaire was designed, based on the research literature and the experiences of the three members of the research team who had received ECT (SC, SPH, LM). Comments on a draft were provided by *Mind*, the United Kingdom's largest mental health charity. There are quantitative questions, with yes/no/don't know, multiple choice, or Likert scale responses; and qualitative questions with open, written responses. The questionnaire asks ECT recipients and family/friends about demographics; the year, country, type and number of ECTs; reasons ECT was given; positive and adverse effects, and their duration; monitoring of cognitive problems; and information given before ECT.

Two of the open questions designed to gather qualitative data (‘Please name up to three positive effects of the ECT’ and ‘Please name up to three negative effects of the ECT’) were asked at the outset to avoid responses being influenced by subsequent questions about specific, named positive and negative outcomes. Participants who responded in the affirmative to later specific questions about memory loss or negative impacts on relationships were asked to give examples in their own words. At the end of the survey, we also asked, ‘Please briefly summarise the impact of ECT on X, in your own words’, and ‘Please briefly summarise the impact of ECT on YOURSELF, in your own words’.

Potential participants confirmed ‘I am at least 18 years old’ and ‘I have either had ECT (but not in the last 4 weeks) or am a friend or relative with an understanding of the impact of ECT on my friend or relative’. The introduction included: ‘This is your opportunity to share your experiences of this treatment, positive, negative, mixed or neutral.’

### Procedure

The questionnaire was disseminated via the online survey tool Qualtrics from January to September 2024. Multiple mental health organizations in multiple countries, on five continents, were contacted. For example, the 44 national mental health groups listed as members of Mental Health Europe (www.mentalhealtheurope.org) were emailed the advertisement and link, with a request to disseminate them to their members and other mental health groups in their countries. In the United Kingdom, the mental health charity Mind disseminated the survey via local branches and members with experience of mental health problems. The survey was also disseminated on social media.

### Data analysis

A total of 1211 people returned surveys: 55 of 63 repeat responses (identified by IP addresses) were excluded because the responses were very similar. A further 12 were excluded because of miscellaneous abnormal responses (e.g., ‘straight‐lining’ on at least three questions, such as ticking ‘severe’ to all 25 adverse effects; or more than one relative of the same patient responding). This left 1144 for analysis.

Responses of the 858 ECT recipients are reported elsewhere (Morrison et al., 2025; Read, Cunliffe, et al., 2025, 2026a, 2026c; Read, Hancock, et al., 2025; Read, Harrop, et al., 2026; Read, Johnstone, et al., 2025). This study reports the responses of the 286 relatives and friends.

Responses to the five open‐ended questions were each subjected to a ‘conventional content analysis’ (Hsieh & Shannon, 2005) to develop categories directly from the data and report them without interpretation. ‘Researchers avoid using preconceived categories, instead allowing the categories and names for categories to flow from the data’ (p. 1279). It is, therefore, an inductive, rather than deductive, process. Analysis begins with reading all the data more than once to ‘achieve immersion and obtain a sense of the whole’. Individual quotes (or ‘codes’) are sorted into clusters with shared meaning, which in turn can, if useful, be grouped into overarching categories, with each cluster becoming a subcategory of a category.

All five analyses were conducted by the same member of the research team (see Limitations). The first three analyses (Examples of Anterograde Amnesia, Examples of Retrograde Amnesia, Examples of Impact on Relationships) were grouped into only 4, 4 and 6 clusters, respectively, and did not require subcategories. The other two analyses (Impact on Patient, Impact on Self) produced nine and ten clusters respectively, which were subsumed under three predetermined overarching categories in both instances (Positive, Negative and Mixed). Multiple examples are provided, verbatim (i.e., with typographical and grammatical errors).

## RESULTS

### Sample characteristics

#### Demographics

The 286 respondents comprised 216 relatives and 70 friends, from 22 countries. Most (86%) of the ECT recipients concerned received their most recent ECT in the United States (37%), the United Kingdom (28%), Australia (6%), Spain (5%), Canada (4%), Denmark (2%), Ireland (2%) or New Zealand (2%). The 14 other countries, providing between one and three respondents, were: Belgium, Brazil, Bulgaria, France, Germany, Guatemala, Hungary, Italy, Netherlands, Norway, South Africa, Sweden, Turkey and Uruguay.

The majority of ECT recipients were white (89%), and most were female (68%). The average age at the time of last ECT was 41.7, with a range of 15–87. Most had their first and last courses of ECT between 2010 and 2024 (57% and 59%, respectively). The most common relatives were daughters (19.4%), mothers (14.8%), sisters (10.6%), sons (6.0%), fathers (3.9%) and brothers (3.9%).

#### Number and type of ECTs

Of the 222 responding to the relevant question, 47 (21.2%) of the ECT recipients had only one series of treatments, 79 had between 2 and 5 (35.6%), 35 between 6 and 10 (15.8%) and 61 (27.5%) ‘had more than 10’. Of the 193 people responding to the relevant question, 44 (22.8%) ECT recipients had received between one and five ECTs in the most recent series, 79 (40.9%) between 6 and 10, 40 (20.7%) between 11 and 20 and 30 (15.5%) ‘more than 20’. Of the 109 who remembered being told which electrode placement was used, 80 (73.4%) reported bilateral and 29 (26.6%) unilateral.

#### Reasons for ECT

Of the 271 who responded to a question about why ECT was prescribed, 189 (69.7%) selected ‘depression’, 58 (21.4%) selected ‘psychosis/schizophrenia’, 55 (20.3%) selected ‘bipolar disorder/mania’ and 26 (9.6%) selected ‘catatonia’. Of the 24 who offered an ‘other’ reason, three said PTSD.

### Efficacy

Efficacy was assessed in five ways. First, when asked ‘How would you describe the problems/symptoms for which ECT was prescribed, just after the end of the treatments, compared to just before?’, 45.1% reported some degree of improvement, and 42.4% thought ECT had made the problem worse to some degree (see Table 1). The modal response (19.4%) was ‘very much worse’.

**TABLE 1:** Efficacy of ECT.

	Effect of ECT on the problem it was prescribed for (*n* = 248)		Effect of ECT on X's overall quality of life (*n* = 251)	
Very much worse	48	19.4%	79	31.5%
Much worse	36	14.5%	48	19.1%
Minimally worse	21	8.5%	26	10.4%
No change	31	12.5%	17	6.8%
Minimally improved	41	16.5%	23	9.2%
Much improved	34	13.7%	22	8.8%
Very much improved	37	14.9%	36	14.3%

Abbreviation: ECT, electroconvulsive therapy.

Second, when asked, ‘How did ECT affect X's overall quality of life?’ 32.3% reported some degree of improvement, and 61.0% said it was made worse to some extent. The modal response, again, was ‘very much worse’ (31.5%) (Table 1).

Third, respondents were asked, ‘During the treatment, what effect did ECT have on X's mood?’ Table 2 shows that 41.9% reported some degree of improvement in mood, and 33.9% thought mood had been made worse to some extent. The modal response (24.2%) was ‘no change’. Of those who reported some mood improvement, 31.3% thought that the improvement lasted less than 3 months. So, overall, 28.5% (67/235) reported some mood improvement lasting 3 months or more.

**TABLE 2:** During the treatment, what effect did ECT have on X's mood? (*n* = 237).

Much better	53	22.0%
Somewhat better	47	19.9%
About the same	57	24.2%
Somewhat worse	34	14.4%
Much worse	46	19.5%

Abbreviation: ECT, electroconvulsive therapy.

Fourth, participants were asked ‘Overall, how helpful was ECT?’ The modal response (60.1%) was ‘not at all’ (see Table 3).

**TABLE 3:** Overall, how helpful/harmful was ECT?

	Overall, how helpful was ECT? (*n* = 248)		Overall, how harmful was ECT? (*n* = 251)	
Very	52	21.0%	134	53.4%
Somewhat	26	10.5%	45	17.9%
Slightly	21	8.5%	24	9.6%
Not at all	149	60.1%	48	19.1%

Abbreviation: ECT, electroconvulsive therapy.

#### Suicidality

Finally, of the 214 who answered ‘During the treatment, what effect did ECT have on how suicidal X felt?’ (with three response options), 35.0% reported that ECT made X ‘less suicidal’ and 19.2% selected ‘more suicidal’. The modal response was ‘no difference’ (45.8%).

### Adverse effects

When asked ‘Overall, how harmful was ECT?’ the majority (81.9%) reported it was harmful to some extent, with the modal response (53.4%) being ‘very harmful’ (see Table 3).

#### Spontaneous reporting of memory loss in general

Participants were asked to name up to three positive effects of the ECT and up to three negative effects. Of the 214 family/friends who reported negative effects, 145 (67.8%) referred to ‘memory loss’, ‘memory problems’, ‘forgetting’, etc. Of the 118 family/friends who reported positive effects of ECT, only one cited improvement in memory. Two mentioned losses of memory for ‘trauma’ or ‘pain’ as a positive effect.

#### Comprehensive Psychopathological Rating Scale

The CPRS defines a change in memory functioning as a difference of at least two of seven scale points between two assessments, made before and immediately after treatment (Sigström et al., 2020) (see Table 4). According to the ratings of the relatives/friends, 50.9% deteriorated (by two or more points), 38.6% were unchanged and 10.5% improved (by two or more points). The mean score after ECT (3.47) was significantly worse than the mean before ECT (1.95) (*t* = 8.75, df = 219, *p* < .001).

**TABLE 4:** How would you describe X's memory overall? just before and just after the last series of ECT?

Score	Descriptor	Before ECT (*n* = 228)	After ECT
0	Memory as usual	89 (39.0%)	26 (11.7%)
1		14 (6.1%)	17 (7.7%)
2	Occasional memory lapses	48 (21.1%)	24 (10.8)
3		14 (6.1%)	19 (8.6%)
4	Socially inconvenient or disturbing loss of memory	34 (14.9%)	65 (29.3%)
5		18 (7.9%)	38 (17.1%)
6	Complete inability to remember	11 (4.8%)	33 (14.9%)
	Mean (standard deviation)	1.95 (1.94)	3.47 (1.89)

Abbreviation: ECT, electroconvulsive therapy.

#### Anterograde amnesia

A total of 211 family/friends responded to ‘What effect did ECT have on X's ability to recall things that have just happened?’ (see Table 5). The majority (60.7%) reported that anterograde amnesia was made either ‘somewhat worse’ or ‘much worse’ by ECT. Improved capacity was reported by 9.0%. The modal response (32.7%) was ‘much worse’.

**TABLE 5:** Anterograde and retrograde amnesia.

	Anterograde amnesia (*n* = 211)	Retrograde amnesia (*n* = 225)
1 Much worse	69 (32.7%)	102 (45.3%)
2 Somewhat worse	59 (28.0%)	63 (28.0%)
3 About the same	64 (30.3%)	43 (19.1%)
4 Somewhat better	8 (3.8%)	10 (4.4%)
5 Much better	11 (5.2%)	7 (3.1%)
Mean (SD)	2.21 (1.10)	1.92 (1.05)

Respondents were asked how long this reduced capacity lasted. (To enable meaningful analysis, respondents who had their first ECT in 2021 or later were excluded). Ninety‐one of the family/friends who reported some degree of reduced capacity to retain new information also answered the question about how long the reduced ability lasted. The modal response (56%) was ‘more than 3 years’ (12.1% responded 4 weeks or less, 23.1%– 1 to 12 months and 8.8%– 1 to 3 years).

Ninety‐four people gave one (15) or two (79) examples of their relative/friend being unable to retain new information. Table 6 provides some examples from the content analysis.

**TABLE 6:** Examples of anterograde amnesia (*n* = 94).

Events that happened earlier that day	21	‘What she ate at a previous meal’ ‘She sometimes didn't know where she had been that day’ ‘Forgot that we just went grocery shopping’
Interactions with family/friends	18	‘Birth of friends baby’ ‘Just this month, 4 years post ECT, didn't remember his brother had helped him move 6 months prior’ ‘She did not recognize me’ ‘Couldnt remember we'd had intercourse the next day’
Difficulties in conversations	16	‘He said his train of thought was disturbed as he spoke, he forgot what he was saying in the middle of speaking’ ‘In conversations he has to have people repeat things. Loses train of thought’ ‘She got lost in conversations, so her wonderful conversationalist abilities were butchered’
Other	29	‘Working memory issues when problem solving’ ‘Cannot remember directions to things and events without writing them down’ ‘Forgot what doctor said at appointment’ ‘Can't retrieve words as readily’

#### Retrograde amnesia

A total of 225 family/friends responded to ‘What effect did ECT have on X's ability to remember events in their life that happened before their ECT?’ (see Table 5). The majority (73.3%) reported that retrograde amnesia was made either ‘somewhat’ or ‘much’ worse by ECT. The modal response (54.6%) was that ECT had made the capacity to remember pre‐ECT events ‘much worse’. Improved capacity as a result of ECT was reported by 7.5%.

A total of 117 family/friends who had ECT for the first time in 2020 or before reported some degree of reduced memory for past events and reported how long the reduction lasted. The modal response, again, was ‘more than 3 years’ (70.1%). 6.0% responded 4 weeks or less, 15.4% responded 1 to 12 months, and 8.5% responded 1 to 3 years.

A total of 133 respondents gave one (13) or two (120) examples (see Table 7).

**TABLE 7:** Examples of retrograde amnesia (*n* = 133).

Child birth and development	29	‘Birth of youngest child several months prior to ECT’ ‘Birth of son and other events of the year’ ‘Children as babies’ ‘Birth of three girls and early years’ ‘He couldn't remember much about his son's first year of life which was before his first ECT treatment’
Other important family events	29	‘Memorable events like family vacations’ ‘Death of her father’ ‘Arrival of grandchildren and great grandchildren’ ‘Meeting husband and wedding’ ‘Birthdays and anniversaries (had a perfect recollection before)’ ‘They don't remember any part of my wedding that they were in’
Patients' own childhood	27	‘Childhood events we shared’ ‘They don't remember so many instances of our childhood together’ ‘No memory of high school’ ‘Most childhood memories were gone’ ‘Elements of early life trauma’
Other	48	‘Living in another country for a few years’ ‘Completely forgot the entire year before/after the two series of ECT, and never again recollected much of it.’ ‘Recovered most of his cognitive ability and memory after a few years, but persistent losses of substantial “patches” of his past, usually particular events or time periods’ ‘Events around being committed and forced to have it’ ‘Cannot remember skills, even basics of skills, previously mastered and skilled’ ‘He didn't remember his professional education and training well enough to remain employed in his field’

#### Other adverse effects

Table 8 lists the reported incidence and severity of 25 other adverse effects. A total of 21 of the 25 were reported to have occurred, to some extent, by 50% or more of participants, most frequently: Difficulty concentrating (79.1%), Fatigue (73.3%), Emotional blunting (72.6%), Losing train of thought (71.5%), Loss of independence (71.5%) and Relationship problems (70.2%).

**TABLE 8:** Incidence and severity of adverse effects reported by relatives and friends of ECT recipients.

	*N*	1. Not at all	2. Mild	3. Moderate	4. Severe	Mean
Difficulty concentrating	191	20.9%	15.2%	22.5%	41.4%	2.84
Emotional blunting	175	27.4%	11.4%	22.3%	38.9%	2.73
Losing train of thought	174	28.5%	15.1%	18.0%	38.4%	2.68
Loss of independence	172	28.5%	15.1%	18.0%	38.4%	2.66
Fatigue	176	26.7%	14.8%	23.9%	34.7%	2.66
Relationship problems	171	29.8%	14.6%	18.7%	36.8%	2.63
Difficulty navigating	162	32.1%	16.0%	19.8%	32.1%	2.52
Loss of a job	157	45.2%	5.1%	8.3%	41.4%	2.46
Difficulty driving	152	35.5%	15.8%	17.8%	30.9%	2.44
Difficulty reading	166	46.4%	24.1%	15.7%	13.9%	2.44
Difficulty cooking	166	32.5%	19.3%	23.5%	24.7%	2.40
Difficulty using the computer	154	39.0%	14.3%	22.7%	24.0%	2.32
Difficulty shopping	154	39.0%	14.3%	22.7%	24.0%	2.26
Difficulty using money	163	36.2%	27.0%	18.4%	18.4%	2.19
Loss of vocabulary	166	36.7%	23.5%	25.9%	13.9%	2.17
Headaches (other than when waking up from ECT)	89	40.4%	22.5%	19.1%	18.0%	2.15
Sensitivity to noise	154	48.1%	20.8%	11.0%	20.1%	2.03
Slurred speech	156	44.9%	24.4%	16.0%	14.7%	2.01
Difficulty recognizing faces	166	46.4%	24.1%	15.7%	13.9%	1.97
Falling over	163	56.4%	15.3%	16.6%	11.7%	1.83
Sensitivity to light	149	61.1%	12.8%	11.4%	14.8%	1.80
Shaky hands	153	47.7%	19.6%	19.0%	13.7%	1.67
Walking into things	144	64.6%	16.7%	9.7%	9.0%	1.63
Seizures/convulsions	146	76.7%	8.9%	6.2%	8.2%	1.46

Abbreviation: ECT, electroconvulsive therapy.

Seven adverse effects were reported to be ‘severe’ by at least one in three respondents, most frequently: Difficulty concentrating (41.4%), Loss of job (41.4%) and Emotional blunting (39.9%). When asked to name any other adverse effects, the most cited were Increased or new suicidality (8), Pain (4) and Anxiety/nervousness (4).

#### Effects on relationships

A total of 209 responded to ‘Has memory loss or any of these other side effects negatively impacted X's relationships with family or friends?’ 65.1% responded ‘yes’, 23.9% said ‘no’ and 11.0% were ‘not sure’. When those who said ‘yes’ were asked to ‘Describe the negative impact on X's relationships with family or friends’, 116 did so. Table 9 provides some examples. The most common type of impact was memory‐related, followed by emotional blunting.

**TABLE 9:** Examples of reported impact of ECT on relationships (*n* = 116).

**Memory related (23)**
It's hard to talk to my mum about things from me growing up, positive or negative memories, because to hear them say they don't remember is hard and awkward
Its hard to talk about things he doesn't remember, reducing the amount of time we talk to him
He had a lot of pride in his intellect and memory, and his inability to recall common events that relatives and friends did created shame and made him less social
People would be offended that she didn't remember a date or outing the week before
I lost my friend. He did not remember me
**Emotional blunting (15)**
Loss of emotions. Numbness. Not so communicative like before the ECT.
It has impacted our relationship. This empty checked out way of being has really increased after the last 6 ECT sessions and it's a struggle to have a conversation.
Extreme emotional blunting to a degree of being unable to form or maintain relationships
Dad was like a zombie after ECT
**Divorce (11)**
We were divorced because of the trauma of ECT
His wife gave him a surprise divorce; years of being a single parent and parenting him in his needy, vegetative state naturally pushed her over the edge
**Irritable/Aggressive (11)**
Snapping or lashing out at people
He screamed and yelled at us. We weren't bad kids, he just couldn't take noises
Agitation and frustration, along with increased irritation contribute to interpersonal conflict and social withdrawal
**Problems conversing (10)**
Loss of memory made conversation more difficult
Less contact b/c she couldnt hold a coherent conversation
**Friends withdrawing (9)**
Many friends left
Ostracized from family and friends as they did not understand her unusual behaviours after ECT

Abbreviation: ECT, electroconvulsive therapy.

#### Brain damage

A total of 155 responded to ‘Has X suffered brain damage as a result of ECT?’. 34.2% said ‘yes’, 18.1% said ‘no’ and 47.7% were ‘not sure’.

### Coercion

Of the 221 who answered the question, ‘Did X have ECT voluntarily or were they forced or pressured to have it?’ 104 (47.1%) selected ‘voluntarily’, 70 (31.7%) ‘gave consent under pressure’ and 47 (21.3%) ‘without consent/against their will’.

### Recommendations

Of the 218 responding to the relevant question, one in four (24.9%) would ‘recommend ECT to a friend or relative if a psychiatrist thought they needed it’ (66.8% said ‘no’ and 8.3% were ‘not sure’). Slightly fewer (22.5%) said ‘yes’ to ‘Would you want to have ECT yourself, if a psychiatrist thought you needed it?’ (72.5% said ‘no’ and 5.0% were ‘not sure’).

### Overall impact on the ECT recipient

Of the 173 who responded to the open question, ‘Please briefly summarise the impact of ECT on X, in your own words’, 41 (23.7%) reported positive impacts, including 17 reports using superlatives such as ‘amazing’ ‘excellent’ ‘dramatic’ ‘huge improvement’ etc., plus 13 references to ECT saving the person's life (7.5%). Fifteen (8.7%) told of mixed impacts, typically short‐term benefits followed by longer‐term harms.

A total of 117 (67.6%) described negative impacts. Content analysis found that the most common types of negative impacts reported were: extreme or permanent disability (51), memory loss/cognitive impairment/brain damage (48), job loss (14), increased depression/suicidality (14), impaired relationships (14) and trauma (11|). Eighteen of the reports were expressed in strong or angry terms. Table 10 lists examples of the positive, mixed and negative reports, with more examples provided for the larger categories. One person reported no impact (‘no impact / in the end she fled from the treatment room, so this treatment was stopped’) and one report was ambiguous (‘life changing’).

**TABLE 10:** Examples of responses to ‘Briefly summarise the impact of ECT on X, in your own words’ (*n* = 173).

**POSITIVE** (41)
**Significant/extreme benefits (17)**
Very positive. It turned her from someone who would need total care possibly for the rest of her life to someone who could function as a normal adult
It was a miracle. He went from not eating or drinking, not acknowledging anyone around him to being himself again
It helped him recover fully after being in hospital for months severely unwell
Significant improvement in quality of life
Amazing treatment—dramatic improvement when nothing else worked
**Life‐saving (13)**
This was a life saving treatment, with a huge improvement in their mood, ability to engage with friends and family, hugely improved quality of life
Got them back, would have died otherwise
It saved her life
**MIXED (15)**
Overall improved mental health, however lasting memory and health problems
The first course of treatment had an immediate beneficial effect, eliminating most of the symptoms of extreme anxiety, depression & psychosis. The second course of treatment, 10 years later, had no discernible benefit, but caused lasting damage to his memory which, in turn, only exacerbated his existing symptoms
At first, it seemed like a miracle, as X was no longer comatose. Eight years later, it was recognized as a disaster with loss of so many quality of life issues
Temporary improvement in emotional stability, long‐term destruction of memory and cognitive ability such as learning
It stopped her continuing attempts to commit suicide, eventually, but probably only because she was so brain damaged by it
**NEGATIVE (117)**
**Extreme/permanent disability (51)**
A life completely ruined and devastated. Utter invalidity
She stopped being independent, she had to be taken care of, she, sadly, was like a plant
She was permanently disabled by the forced ECT
My beautiful, brilliant, motivated sister‐in‐law often cannot talk at all. She no longer can walk. She cannot prepare herself food. She is so courageous. She does not remember her childhood. She can show you a photograph and tell you what someone has told her about that childhood moment
It was irreversibly life‐damaging, both physically and emotionally.
It was extremely life altering and destroying. It was heartbreaking to witness
I saw my beautiful radiant creative multi‐talented mother: scientist, activist, poet and writer, community leadership, in addition to all the above, she played guitar and piano and taught folk dance, become a withdrawn, fearful, dependent person who, for many years, looked at her feet when she walked, so that she would not lose her balance or fall. It was like they crushed a beautiful flower
It destroyed his life
As a psychiatrist knowing what I know about X she was ‘misdiagnosed’ with bipolar following severe trauma in her early life, she was never given proper therapy and heavily medicated and ECT. Ultimately this resulted in a loss of independence which she will never get back
Devastating, her life will be forever negatively impacted and she likely will be battling the after effects on her health for the rest of her life.
Completely left her disabled, needs a wheelchair, often stops breathing when stimuli is overpowering, her cognition is diminished, her memory is gone
**Memory loss/cognitive impairment/brain damage (48)**
She has severe memory loss and couldn't work for a few years
The damage was just to much to bear. The constant headaches and brain damage
Really bad short term memory but long term has been affected too. Becomes very confused and illogical. My Granddad is very numb, emotionless
Loss of confidence, cognitive ability, and memory has been lasting and distressing
I feel it has still impacted memory 6 years later which had impacted job performance and organizational skills. Loss of two jobs. Less motivation. Increase in substance abuse and inability to maintain relationships
She has lost a large chunk of her memory, can't remember a large period of her life. She has a very high IQ and an amazing memory, so for her to lose part of it is very hard for her
Complete, permanent memory loss of the entire year surrounding the ECT; major long‐term memory losses, though most of these, not all, did come back after a few years in part due to cognitive/memory practicing and efforts by X
This left her brain damaged and with constant crippling headaches (she is now the 2nd ECT victim I know who has this)
He could't remember anything to hold a job or keep his marriage. He tried to tell doctors that he felt like he had brain damage. His ability to perform tasks that required executive function was completely gone for years
**Strong/angry statements (18)**
Its disgusting and trechorous treatment of a fellow human being
Feels violated and treated against his will. Depression can be recovered from, brain damage not so
It is immoral and unethical what was done to her
I believe ECT was an assault on X in mind and body and in her essence or spirit
Brutal and inhuman
Barbaric
Disgusting and cruel
Those prescribing or preforming ECT should be jailed for abuse, and have all medical licences revoked
**Job Loss (14)**
Compromised his ability to hold a job and is still impacting his ability to work as it has affected his memory and cognition
The loss of his job that he had worked so hard to attain with his schooling that he had spent time and money for
Loss of job, income, driver's licence. Loss of social confidence. Loss of purpose
**More depressed/suicidal (14)**
She was more suicidal, not less, and was dramatically less functional because of memory and cognitive damage. It closed off her ability to benefit from other paths to healing. The doctor was an “ECT specialist” and did not seem capable of taking into account the effect of the many losses and stressors she was experiencing
I don't think it's all that safe, because it made my cousin more depressed and suicidal
He became fearful and lacked interest in things he used to enjoy because he could not remember how to do them and he lost interest in life and tried to commit suicide 2 years later
He feels more hopeless and helpless and he has spoken even more about suicide since the last bout of ECT
**Impaired relationships (14)**
After, he seemed to barely tolerate us or any company
She lost her intelligence, independence, her relationships, and her place in this world
Ruined his life totally. No relating, no family life
It ruined his marriage
It caused isolation from society, she was a social sciences teacher and her teaching abilities, relationships with students and families of students were significantly destroyed
I was divorced. It led to the demise of my marriage. I lost my best friend
**Traumatized (11)**
She was traumatized by the ECT and felt it was yet another form of male control and abuse where she had no voice
Xxxx was devastated by how ECT changed her. After, she lived in a constant state of terror
He would beg and scream not to be shocked
She has ongoing trauma from ECT

Abbreviation: ECT, electroconvulsive therapy.

### Impact on relatives/friends

A total of 135 responded to the final question of the survey: ‘Please briefly summarise the impact of X having ECT on YOURSELF, in your own words’. 28 related positive impacts on themselves, mostly (10) about getting the person back. Nine reported mixed impacts. Ninety‐eight reported negative impacts on themselves, most commonly about sadness (26) or distress (17) from the adverse effects. Seventeen wrote about increased caretaking. Table 11 offers some examples.

**TABLE 11:** Examples of responses to ‘Briefly summarise the impact of X having ECT on YOURSELF in your own words’ (*n* = 135).

**POSITIVE (28)**
**Got them back (10)**
Wonderful. I got my husband back. Before ECT he just sat in the chair & I could get nothing out of him
It helped me get my auntie back. She was very ill and confused. The ECT helped her recovery over about a month and without it she would have died
ECT has given me my friend back
**Relieved (4)**
Such a relief to see effectiveness
Life changing. I'm no longer living in constant fear of something terrible happening
**Grateful (4)**
I was so grateful to the doctors and healthcare team that saved his life.
I was gratified the health service provided the ECT treatment
**MIXED (9)**
Feelings of gladness that something somewhat alleviated her suffering. Feeling of anger that she did not have access to the wider range of supports that exist for distress.
I was happy to see life again on her face, but this faded away very fast.
I am glad to see her out of hospital, but it saddens me that she cannot think clearly and that her autonomy has been so reduced
**NEGATIVE (98)**
**Sadness/loss (26)**
That blank vacant stare breaks my heart
Feeling lonely because husband and I no longer share many memories
I have always mourned the loss of my father's joyful personality
I was heartbroken for her, and for myself, and I was startled and worried to see the bizarre effects on her
I lost my friend
**Distressing to witness negative effects (17)**
It's been very hard to see my Granddad (who raised me) go from very intelligent, outgoing, easy going, caring, funny, sociable, independent, sensitive and emotional to the shell of a person he is now. He has no feelings, no emotions, he is now very stubborn, very confused with no logic, little understanding of anything within day to day life. Always looks lost and sad so it is hard being in his company
It was difficult to watch our outgoing son become so lacklustre in his personality. …and experiencing his attempt at suicide in our home certainly impacted us
Very difficult to see as a parent
Traumatising for a young child to see their mother like that and for her to be unable to remember significant life events
It was one of the hardest things I have ever had to witness
It's caused me huge distress seeing my father deteriorate and become a shadow of himself
**Caretaking (17)**
I will need to support her for the rest of her life due to the impact ECT has had on her
Years of trying to diagnose and find solutions to the problems she has had as a result of ECT have taken its toll on me physically, emotionally and financially
Since we met, much of my time has been invested in his recovery and rehabilitation. We've been able to make a lot of progress in terms of his quality of life
Our care bills are astronomical and myself and siblings all have mild depression too as a result
I was forced to become her caregiver in order to protect her from further abuse by ECT/Psychiatrists
**Terrifying/frightening/traumatizing/shocking (14)**
How did her ECT impact me? It's terrifying. I feel helpless I was 12 and it was a terrifying experience for me to witness her in a state of confusion and then to have her come home and as my sole carer be confused and forgetful about me and my needs
A nervous wreck. Almost had a heart attack and tons of anxiety. Couldn't sleep eat think. Frantically making calls to find out how to stop this abuse
It's traumatizing. I can still hear the fear in her voice
I was terrified by so much cruelty
He died. It was more than terrible. Basically awful
**Guilt (11)**
I feel guilty that I wasn't able to stop it. I feel like I let her down. I was supposed to protect her
Guilt that I did not research and prevent it
They didnt speek to me for over a year, was very angry at me for not being an advocate for them I did what I thought was best and tried to be supportive but it wasn't what they needed. I didn't know how to do what was needed for them although I thought I was, I failed
Decades later I am still devastated that I did not or could not prevent it from happening, that I didn't understand enough at the time, was not forceful enough to help him fight more legally to prevent it
As a parent, I suffer from guilt and regret that my decision to allow my child to undergo ECT was wrong
**Difficulties interacting (10)**
I was so sorry and terrified to see my lovely friend losing words to talk day after day, or forgetting what we have shared together. She was a clever woman before ECT
We no longer communicate and we used to often
She was not compassionate and her emotions were blunted. She could not understand another's viewpoint
**Anger/loss of trust in medical professionals (9)**
After initially trusting the medical judgement of the psychiatrist I researched it in some detail and was appalled by the non scientific nature of the intervention
Serious loss of faith in the medical community at large. Great concern that Drs. prescribe this treatment to make big money for hospitals and themselves and not to actually help the patients
It forever changed my views of the mental health system, and of forced treatment, and of psychiatrists—making me have no respect for them, and a concern and fear of their extraordinary legal powers
I am very angry that I was misinformed and misled about the harm that ECT does and its effectiveness

Abbreviation: ECT, electroconvulsive therapy.

### Deaths

Fifty‐three of the ECT recipients were dead. The most common causes cited were suicide (8), old age (7), heart attack (5) and stroke (4). When the relatives/friends of the 53 were asked ‘Do you believe ECT had any role in X's death?’, eight (15.1%) thought ECT was ‘a direct cause’ of the death, and 19 (35.8%) thought it was ‘a partial/indirect contributor to the death’.

In four of the eight suicides the relative/friend thought ECT was a ‘partial/indirect contributor’. One wrote:

I believe ECT contributed to her death by worsening her already difficult circumstances. She had been struggling for 2 years with untreated postpartum depression and prolonged side effects from Zoloft while raising a baby without support from her family. Her family and psychiatrist pressured her into continuing ECT. When she gave in, ECT did not address her issues, provided no benefits, and caused brain damage. Waking up to a world distorted by brain damage is terrifying, especially when no one believes you and there's no guidance on how to recover.

Another commented

I believe that ECT robbed X of access on an emotional level to the traumatic events that precipitated her mental health crisis, that is the death by suicide of my father 3/4 months before the ECT and the coercive control inside the family exerted by our mother. I believe that had X had been offered the support of psychotherapy or counselling to help her process these issues rather than numbing levels of psychiatric medication and ECT based on her psychiatrists diagnosis that X's depression was purely genetic / endogenous and not related to the death of her father by suicide and dynamics inside the family, I believe the outcome for X could have been different and she could have recovered in time rather than taking her own life.

Four of the eight deaths described as being directly caused by ECT occurred several years after the last treatment. The other four are described in Table 12.

**TABLE 12:** ECT‐related deaths.

Country	Age at last ECT	Year of last ECT	‘Why do you think ECT caused X's death?’
Denmark	76	2022	Died the day after ECT. Declared braindead from a brain bleed caused by ECT. From the records, several mistakes were made
Guatemala	40	1998	He died because of it in Guatemala. I am certain. His brain was fried. He could no longer breathe. He was disconnected from an artificial breather
Norway	?	?	Committed suicide almost immediately after end of treatment. Severe brain damage, trauma, PTSD, gaslighting surrounding the treatment, coercion, abuse by psychiatrist
United States	45	2018	Had a brain hemorrhage on her first ECT. She entered perpetual seizures (status epilepticus) while at the outpatient clinic, Had emergency brain surgery within 1 h of going under. The brain bleeding led to 2 heart attacks due to pressure on her brainstem, plus craniectomy to relieve pressure. CPR. Coma for nearly 2 weeks, and digestive blockages. Finally her 18 year old daughter made the decision to let her go after she'd been on life support for 2 weeks

## DISCUSSION

Our findings have, as hoped, given a voice to the largest number of relatives and friends of ECT recipients ever surveyed, on a wide variety of topics and experiences.

### Efficacy

The findings about efficacy closely match the responses of the patients to the same survey (Read, Johnstone, et al., 2025), suggesting a degree of inter‐rater reliability. For example, 45% of both patients and relatives/friends thought that ECT had improved the problem for which it was prescribed. 45% of relatives/friends and 42% of patients thought that mood had been improved. 61% of relatives/friends and 62% of patients reported that ECT had made their quality of life worse. 60% of relatives/friends and 58% of patients thought that ECT was ‘not at all helpful’ overall. The differences in responses to the various questions suggest that outcomes depend on the breadth of the question used to evaluate efficacy. Focussing just on symptoms may indicate higher efficacy than asking about overall effects on wellbeing, which include the negative effects, the price paid for the positive effects (for some).

While it may seem strange to have such an extreme range of responses to each of the efficacy questions, this is predictable for a treatment with no dosing consensus protocols and, therefore, hundreds of possible doses from which to choose (Bergsholm & Bjølseth, 2022). A leading UK ECT proponent has acknowledged, more than 80 years after the first ECT, that ‘the best way to administer ECT with regard to optimizing therapeutic benefit and simultaneously minimizing cognitive side‐effects is not known’ (McLoughlin, 2018). Variable outcomes are, therefore, inevitable.

The reports of both relatives/friends and patients stand in stark contrast to the 80% (or similar) efficacy claims in many ECT information leaflets (Harrop et al., 2021; Read, Morrison, & Harrop, 2023).

### Adverse effects

The very high rates of memory loss and the other adverse effects, that receive even less research attention, are of grave concern. Patients tended to report even higher rates than the relatives/friends (Read, Hancock, et al., 2025). For example, retrograde amnesia was reported by 73% of relatives/friends but 80% of patients; while anterograde amnesia was reported by 61% of relatives/friends but 70% of patients. In our survey, 60% of family/friends (and 61% of patients) reported deterioration on the CPRS. This contrasts sharply with three studies where psychiatrists(including those administering the ECT) made the assessments, which reported deterioration rates of 26% (Brus et al., 2017), 16% (Sigström et al., 2020) and 10% (Royal College of Psychiatrists, 2023).

Short‐term memory loss is usually acknowledged, but longer‐term or permanent damage is often minimized or denied (Harrop et al., 2021; Read, Harrop, et al., 2026; Read, Morrison, & Harrop, 2023). It is important to note that 56% stated that the reported anterograde amnesia lasted more than 3 years, and that 70% of the retrograde amnesia lasted the same period. The corresponding figures for patients were, again, even higher, at 65% and 81% (Read, Hancock, et al., 2025).

An exception to the pattern of even higher rates of adverse outcomes being reported by patients is the finding that 32% of relatives/patients thought that ECT had caused brain damage, a view held by 28% of patients.

The hundreds of patients, relatives and friends reporting such high levels of severe, often permanent, cognitive dysfunction are not atypical and should not be ignored. A meta‐synthesis of 16 qualitative studies of ECT recipients (Wells et al., 2020) found:

In 10 papers, participants reported the loss of other cognitive abilities such as comprehension, ability to communicate and “intellectual ability”. Participants described feeling “stupider” or like a “vegetable”. Some described this as “brain damage”. Three studies reported loss of creativity, artistic ability and imagination.

A large, prospective study concluded that ‘adverse cognitive effects can persist for an extended period, and that they characterize routine treatment with ECT in community settings’ (Sackeim et al., 2007). A New Zealand government report stated ‘ECT may permanently affect memory and sometimes this can be of major personal significance’ and noted the ‘slowness in acceptance by some professional groups that such outcomes are real and significant in people's lives’ (Ministry of Health, 2004, p. 16). A USA manufacturer of ECT devices used worldwide has been required by the FDA to add a warning that their product can cause ‘permanent memory loss or permanent brain damage’ (Schwartzkopff, 2018; Somatics, 2018, p. 4).

### Deaths

If we exclude the 19 deaths that relatives thought were only ‘partially’ caused by ECT, and the four where ECT was considered the ‘direct cause’ but the deaths had occurred well after the ECT, we are left with four cases. This resulting rate of one in 72 (4/286), while of concern, cannot be taken to be an accurate estimate of ECT‐related deaths in general.

The friend of one of these four, the woman who died in the United States, added:

They always suspected it had caused the death because she was taken by ambulance from the clinic to the hospital. But it wasn't until 5.5 years afterwards that the sister‐in‐law joined an online support group to share her family's experience with people considering ECT that someone posted a photo of a page from the APA ECT textbook that said during ECT, the blood‐pressure in the brain spikes 3x normal. It explained the brainstem rupture and hemorrhagic stroke‥ ‥ It still greatly affects her family, especially her children and brother. The hospital held a “hearing.” I put that in quotes because it was a farce‐‐just to close the door on the entire thing. They said the reason she went into perpetual seizures is because she had a “pre‐existing vascular malformation” that ruptured. We never knew of any pre‐existing vascular malformation. I don't even think there were any brain scans done before the ECT. They certainly weren't told death was a risk in this “safe” treatment. It all seems like they were just covering themselves. The hospital attorney believed them without any evidence of a pre‐existing rupture. If they knew a brain hemorrhage or death was a possibility, why was that not discussed before? If they knew someone could stop breathing, why didn't the outpatient clinic have equipment like the breathing tube? If they knew status epilepticus was a possibility, why didn't they have all medication and medical equipment on site?

### Overall impact

Perhaps the most important and novel findings are the responses to the open, qualitative questions, where participants were free to say anything they wanted rather than respond to specific questions of interest to the researchers, with pre‐determined response options.

Clearly, some people do feel that ECT had a dramatic positive, sometimes life‐saving, effect on their relative or friend that ‘brought the person back’ to them. But three times as many people reported negative impacts than positive ones. A minority wrote about short‐term benefits and long‐term damage. The majority wrote, often with great emotion (including anger and despair) only about their distress at witnessing the damage done to a loved one by ECT, and about the loss of the person they had once known. Many wrote about the negative effect on their relationship with the person in question, often, but not only, because of the damage to memories old and new. They wrote, too, about their new role of caretaker of a damaged person. A smaller number wrote about their guilt for not having intervened to stop the ECT from happening.

Overall, it can be said that although 45% reported that ECT had improved the problem for which it had been given, many of those reported a high price for that improvement, in terms of multiple adverse effects, damaged relationships and overall quality of life. And almost as many, 42%, reported that the problem itself had been made worse.

The voices of relatives and friends need to be heard, taken seriously and acted upon, if only by ensuring that relatives in the future are better informed when ECT is being considered.

Furthermore, a common riposte to ECT recipients' reports of adverse effects is that they are confusing the effects of ECT with the effects of being depressed. The relatives' accounts can be assumed to be free from this potential bias.

### Limitations

There may have been sample bias towards people whose relative or friend had a negative outcome, because of the dissemination of the survey on social media by the researchers, some of whom have critiqued ECT in research papers and online. To minimize this, social media posts included phrases like ‘Positive, mixed and neutral experiences are all equally valued’.

Responses were based on memory, sometimes for events many years in the past. The responses were also, by definition, ‘second‐hand’, that is, observations of other people, rather than reports of one's own experience (except when the relatives/friends were reporting the impact on themselves). Some of the adverse effects attributed to ECT may have been partially or completely caused by factors other than ECT, such as ageing.

The categorizing of the written answers to the open questions was, inevitably, a subjective process. The ‘trustworthiness’ of the data (Hsieh & Shannon, 2005) was enhanced by the researcher's thorough immersion in the data (see Methods) and by the fact that the analysis was at a manifest rather than latent level (i.e., did not require interpretation of underlying meanings by the researcher). Trustworthiness, however, was potentially diminished by only having one researcher involved, although coding by just one researcher is common, especially for inductive content analysis (Hsieh & Shannon, 2005).

Hopefully sufficient examples have been provided to enable readers to assess whether the categorisation from the content analysis is reasonable.

We could not recruit adequately from countries beyond English speakers from North America, Europe and Australasia without translating the survey into multiple languages, for which we had no resources.

## CONCLUSIONS

At a theoretical level, claims that ECT is a safe and effective treatment are challenged by the experiences of these relatives and friends, as they are by the findings of the recipients themselves (Morrison et al., 2025; Read, Cunliffe, et al., 2025, 2026a, 2026c; Read, Hancock, et al., 2025; Read, Harrop, et al., 2026; Read, Johnstone, et al., 2025) and by previous reviews of the literature (Read & Bentall, 2010; Read, Kirsch, & McGrath, 2019; Wells et al., 2020).

In 2023, a joint report by the World Health Organization and the United Nations (2023) argued that ‘People being offered ECT should also be made aware of all its risks and potential short‐ and long‐term harmful effects, such as memory loss and brain damage’. (p. 58). The ‘Montgomery ruling’ by the UK Supreme Court states that patients must be warned of ‘all material risks to which a reasonable person in the patient's position would attach significance’, not just what doctors think should be shared (Chan et al., 2017). Our data demonstrates lasting, often severe, consequences on many patients, relatives and friends.

From the point of view of the practical implications of our findings, we must emphasize the fact that informed choice is a basic human right. Our findings make it all the more imperative to acknowledge the limited and highly variable efficacy, and list the full range of risks, when offering ECT so that all patients and relatives are fully informed and can, therefore, legally and ethically give consent. This does not appear to be the case at present (Harrop et al., 2021; Read, Harrop, et al., 2026; Read, Morrison, & Harrop, 2023; Wand et al., 2024).

The experiences of these relatives, along with those of the patients themselves (Morrison et al., 2025; Read, Cunliffe, et al., 2025, 2026a, 2026c; Read, Hancock, et al., 2025; Read, Harrop, et al., 2026; Read, Johnstone, et al., 2025) should inform national and professional guidelines.

## AUTHOR CONTRIBUTIONS

**Christopher Harrop:** Conceptualization; methodology; writing – review and editing; investigation. **Lucy Johnstone:** Conceptualization; investigation; methodology; writing – review and editing. **Sarah Price Hancock:** Conceptualization; investigation; methodology; writing – review and editing. **Lisa Morrison:** Conceptualization; investigation; methodology; writing – review and editing. **Sue Cunliffe:** Conceptualization; methodology; writing – review and editing; investigation. **John Read:** Conceptualization; methodology; writing – original draft; investigation; formal analysis.

## CONFLICT OF INTEREST STATEMENT

Professor Read has been a paid Expert Witness in several ECT legal cases in the USA, Canada and New Zealand.

## Data Availability

The data set is still being used for other papers. It contains multiple highly personal stories, some of which may be identifying. Participants have not given permission for their data to be shared.
